# The Potential of JWH-133 to Inhibit the TLR4/NF-κB Signaling Pathway in Uterine Ischemia–Reperfusion Injury

**DOI:** 10.3390/life14101214

**Published:** 2024-09-24

**Authors:** Nihal Inandiklioglu, Taylan Onat, Kayode Yomi Raheem, Savas Kaya

**Affiliations:** 1 Faculty of Medicine, Department of Medical Biology, Yozgat Bozok University, 66200 Yozgat, Türkiye; 2 Faculty of Medicine, Department of Obstetrics and Gynecology, Yozgat Bozok University, 66200 Yozgat, Türkiye; onat.taylan@gmail.com; 3 Faculty of Science, Department of Biochemistry, Adekunle Ajasin University, Akungba 342111, Ondo State, Nigeria; kayoderaheem.y@gmail.com; 4 Faculty of Science, Department of Chemistry, Cumhuriyet University, 58140 Sivas, Türkiye; savaskaya1989@gmail.com

**Keywords:** gene expression, ischemia–reperfusion, JWH-133, molecular docking, uterus

## Abstract

In recent years, significant progress has been made in understanding the biological and molecular pathways that regulate the effects of ischemia–reperfusion (I/R) injuries. However, despite these developments, various pharmacological agents are still being tested to either protect against or mitigate the damage caused by the IR’s harmful consequences. JWH133 is a CB2R-selective agonist and belongs to the class of Δ8-tetrahydrocannabinol. The present study aimed to determine the in vivo effect of JWH-133 on uterine IR injury via the TLR4/NF-κB, pathway. Female Wistar albino rats (n = 40) were randomly divided into five groups. Three different doses of JWH-133 (0.2, 1, and 5 mg/kg) were administered to the rats. RNA was isolated from uterine tissue samples, and gene expression was measured by RT-PCR using specific primers. The interaction energies and binding affinities of JWH-133 with IL-1β, IL-6, NF-κB, TLR-4, and TNF-α were calculated through molecular docking analysis. The expression analysis revealed that JWH-133 administration significantly reduced the expression levels of IL-1β, IL-6, NF-κB, TLR-4, and TNF-α (*p* < 0.05). Notably, in the 1 mg/kg JWH-133 group, all of the gene expression levels decreased significantly (*p* < 0.05). The molecular docking results showed that JWH-133 formed hydrogen bonds with GLU64 of IL-1β, SER226 of IL-6, and SER62 of TNF-α. This study highlights the molecular binding affinity of JWH-133 and its potential effects on inflammation in IR injury. These results pave the way for future research on its potential as a therapeutic target.

## 1. Introduction

Uterine torsion is defined as a rotation of the uterus by more than 45 degrees around its long axis. Uterine torsion can occur in all age groups during the reproductive period, across all parity groups, and at any stage of pregnancy. The torsion always occurs at the junction between the cervix and the corpus uteri. Numerous abnormalities can occur in uterine torsion and the most common are abnormal fetal presentation, myoma uteri, and uterine malformations. This condition, which has a high fetal mortality rate, is typically diagnosed during surgery [1].

Cannabis, the main active ingredient of which is delta-9-tetrahydrocannabinol, activates two G protein-dependent membrane receptors called cannabinoid type-1 receptor (CB1R) and cannabinoid type-2 receptor (CB2R) [2]. CB2R is predominantly found in immune cells, tonsils, spleen, and testis. Since CB2R is expressed in tissues associated with the immune system, these receptors are also referred to as immunocannabinoid system receptors [3]. CB2R agonists have been suggested for therapeutic use in various peripheral disorders, including inflammation, atherosclerosis, inflammatory bowel diseases, ischemia–reperfusion injury (IRI), renal fibrosis, liver cirrhosis, and in preclinical cancer models [4]. JWH-133 is a selective CB2R agonist. Many studies have demonstrated that JWH133 exhibits anticancer, cardioprotective, hepatoprotective, gastroprotective, nephroprotective, anti-inflammatory, antihyperalgesic, neuroprotective, and immunomodulatory effects [5]. IRI has been associated with the initiation of innate immune responses and enhanced adaptive immunity. A group of receptors involved in the pro-inflammatory response established after reperfusion is Toll-Like Receptors (TLRs) [6]. Experimental knockout models have shown that TLR4-deficient mice are protected from IRIs [7]. Inflammatory activation occurs through the induction of the nuclear factor-kB (NF-kB) pathway, resulting in the production of pro-inflammatory cytokines and chemokines, including interleukin-1β (IL-1β), IL-6, and tumor necrosis factor-α (TNF-α) [6]. Furthermore, research has shown that the anti-inflammatory effects of JWH-133 influence the TLR4/NF-kB pathway in models of IRI, sepsis, or inflammation in various tissues [5,8,9]. In a study by Onat et al. [10], JWH-133 was found to reduce NF-kB immunoreactivity in rats with ovarian IRIs. TNF-α, IL-1β, and IL-6 are key cytokines involved in the inflammatory response during IRI. TNF-α not only mediates oxidative stress that leads to cell damage, but also promotes cell survival through NF-κB induction in human endometrial stromal cells and other organs [11,12].

Molecular docking is a computational technique used to predict and analyze the binding of ligands to a receptor or target protein. The present study investigated the potential of JWH-133, a cannabinoid receptor-2 agonist, to influence the TLR4/NF-kB pathway in a rat model of uterine IRI. To further understand the mechanism of action, a molecular docking analysis was performed to predict the potential binding interactions between JWH-133 and the target proteins (IL-1β, IL-6, NF-κB, TLR-4, TNF-α). Additionally, this is the first study to explain the effect of JWH-133 on uterine IRI using both genetic and molecular docking analyses. In this context, the researchers also aimed to investigate the effect of JWH-133 on the expression of genes involved in the TLR4/NF-κB pathway in uterus IRIs, and to correlate the findings with molecular docking results.

## 2. Materials and Methods

### 2.1. Study Design

In this study, Wistar albino female rats (n = 40, 180–220 g) were supplied from Saki Yenili Experimental Animals Production Laboratory Industry and Trade Co., Ankara, Türkiye. The animals were housed in a controlled environment with a temperature of 24 ± 2 °C and 60% humidity, maintained on a 12 h light–dark cycle. They had access to tap water and standard food. All procedures complied with the Guidelines for the Care and Use of Laboratory Animals, as outlined by the National Institutes of Health (USA) and the Declaration of Helsinki. Six hours following the final administration, the rats were anesthetized with ketamine hydrochloride (50 mg/kg, intramuscular/intraperitoneal and 2% xylazine hydrochloride) and 10 mg/kg intramuscular/intraperitoneal for euthanasia. Euthanasia was carried out using the cervical dislocation method, and uterine tissues were subsequently excised for genetic analysis. Every effort was made to minimize animal suffering throughout the study.

### 2.2. Experimental Model

Given the limited number of studies investigating the biological activity of JWH-133 in rats, this study aims to contribute valuable information on dose determination. To this end, a broad dose range was employed. Nevertheless, the dose range was adjusted to encompass the recommended dosages reported in previous research involving other animal models [10,13]. The rats included in the present study were divided into 5 groups: Control group, Group 1 (IRI), Group 2 (IRI + 0.2 mg/kg JWH-133), Group 3 (IRI + 1 mg/kg JWH-133), and Group 4 (IRI + 5 mg/kg JWH-133). After adequate anesthesia, the abdomen was entered through a 2–2.5 cm incision into the abdominal skin. After the uterine horns were exposed, a vascular clip was placed in the abdominal aorta and under the right infundibulopelvic ligament. Ischemia and reperfusion were carried out for 3 h each. JWH-133 (Cayman Chemical, Ann Arbor, MI, USA) was given intraperitoneally 30 min before reperfusion. Meanwhile, Group 1 was injected with distilled water intraperitoneally. After the process, all animals were sacrificed and the right uterine horn was removed.

### 2.3. Genetic Analysis

The isolation of RNA from the tissues was performed with the RNA kit protocol (RNA Extraction Kit, Macherey-Nagel, Düren, Germany). The obtained RNAs were synthesized as cDNA in accordance with the cDNA synthesis kit protocol (High-Capacity cDNA Reverse Transcription Kit, Thermo Fisher, Waltham, MA, USA). The cDNA concentrations of the samples were measured with the Fluorometer (QFX, Denovix, Wilmington, DE, USA). The cDNA concentrations were equalized as 30 ng/μL in all samples. Suitable primer sets (IL-1β: Rn00580432_m1; IL-6: Rn01410330_m1; NF-κB: Rn01399572_m1; TNF-α: Rn01525859_g1; TLR-4: Rn00569848_m1; Glyceraldehyde 3-phosphate dehydrogenase (GAPDH); Rn99999916_s1; Taqman Gene Expression Assay, Thermo Fisher Scientific, USA), RT-PCR Device (Q2000B, LongGene, Hangzhou, China) and a Master-Mix Kit (TaqMan Gene Expression Master Mix, Thermo Fisher Scientific, USA) were used. The GAPDH gene was used as the control gene. The Cycle threshold (Ct) of each sample was recorded, and the 2^∆∆Ct^ values of the data were analyzed.

### 2.4. Molecular Docking Analysis

#### 2.4.1. Protein and Ligand Preparation

The IL-1β (PDB-ID: 1L1B), IL-6 (PDB-ID: 1l1R), NF-κB (PDB-ID: 4IDV), TNF-α (PDB-ID: 3L9J), and TLR-4 (PDB-ID: 2Z5V) structure was retrieved from protein data bank (https://www.rcsb.org/ accessed on 21 July 2023). The structure was preprocessed using the Protein Preparation Wizard, which is integrated into Schrödinger’s Maestro tool as described by Sastry et al. [14]. To eliminate steric clashes, all water molecules and co-crystal ligands were removed, and the structure was minimized using the OPLS4 forcefield. In addition, Ligprep, another tool embedded in the Schrodinger software SSE4.2 [15], was employed to prepare the compound (JWH-133) for molecular docking studies. The Ligprep tool generated different ionization states and tautomeric forms of the compounds at pH 7.0, following the methodology described by Li, Robertson and Jensen [16].

#### 2.4.2. Receptor Grid Generation and Glide Docking

A grid box was created around the binding pocket of each protein structure before conducting docking studies. The receptor grid generation panel was utilized to generate a grid at the binding sites of the prepared protein. Prepared ligands were then docked within the generated grid using Glide SP docking, a precision tool from Schrodinger software SSE4.2 [17].

### 2.5. Statistical Analysis

Statistical analysis was conducted with the SPSS 22 (IBM Corp. released 2011. IBM SPSS Statistics for Windows, version 20.0, Armonk, NY, USA: IBM Corp.). The normality of the data was tested by Kolmogorov–Smirnov/Shapiro–Wilk tests. The *p*-values were calculated based on a Student’s *t*-test of the replicate 2^(−Delta CT)^ values for each gene in the control and treatment groups. The *p* < 0.05 was considered to be statistically significant.

## 3. Results

### 3.1. Genetic

The expression levels of the IL-1β, IL-6, NF-κB, TLR-4, and TNF-α genes are presented in Table 1 and compared with those of the control group. When the researchers evaluated the results of the expression analysis between the groups, it was determined that the expression levels of all genes in Group 3 (IRI + 1 mg/kg JWH-133) decreased significantly (*p* < 0.05). In addition, the IL-6 and NF-kB genes showed significant results in all groups compared to the Control group (*p* < 0.05) (Table 1).

### 3.2. Molecular Docking

Docking analysis is a computational technique used to predict the binding affinity of small molecules such as JWH-133 to the binding pocket of the proteins. The binding score of the JWH-133 with the binding pocket of each protein (IL-1β, IL-6, NF-κB, TLR-4, and TNF-α) is presented in Table 2. The results of the docking analysis indicate that JWH-133 exhibit a high binding affinity to the TLR-4 protein, with a binding score of −4.0 kcal/mol, followed by IL-1β with a score of −3.99 kcal/mol, and NF-kB with a score of −3.75 kcal/mol. On the other hand, JWH-133 exhibits a relatively weak binding affinity to IL-6 and TNF-α with binding scores of −3.08 and −3.00 kcal/mol, respectively. These findings suggest that JWH-133 can preferentially bind to TLR-4, IL-1β and NF-κB proteins which are involved in immune response regulation and inflammatory processes. The 2D intermolecular interaction between JWH-133 and amino residues occupied at each of the protein binding pockets is illustrated in the Supplementary Materials, highlighting the potential binding sites of JWH-133 with each protein. It was discovered that JWH-133 formed a hydrogen bond with the amino acid residues of glutamic acid (GLU) 64 of IL-1β, serine (SER) 226 of IL-6, and SER62 of TNF-α.

## 4. Discussion

The effects of JWH-133 on the expression of IL-1β, IL-6, NF-κB, TLR4, and TNF-α in the uterine tissue of IR rats were evaluated. Treatment with a 1 mg/kg dose of JWH-133 significantly reduced the expression levels of the IL-1β, IL-6, NF-κB, TLR4, and TNF-α genes. The molecular docking analysis results suggest that JWH-133 can preferentially bind to TLR-4, IL-1β, and NF-κB proteins, which play key roles in immune response regulation and inflammatory processes. Additionally, it was observed that JWH-133 forms a hydrogen bond with the glutamic acid (GLU) 64 residue of IL-1β, the serine (SER) 226 residue of IL-6, and the SER62 residue of TNF-α.

Across the world, approximately one million women are waiting for treatment due to the absence of a uterus or the presence of a dysfunctional uterus. The only reported treatment option for this patient group to become mothers is uterine transplantation [18]. It is estimated that, to date, at least 80 uterine transplant procedures have been performed globally, resulting in more than 40 births [19]. However, IRI during this procedure is associated with graft dysfunction and rejection. Therefore, preventing IR-induced cell damage is crucial for a successful transplant. IRI is a critical condition in which physicians must control cellular damage and preserve organ function, involving a complex series of interconnected cellular and humoral events. In the IRI model, it has been shown that TNF-α expression increases positively in the uterine tissue with the increase in apoptotic cells [20]. Similarly, Liu et al. [21] found an increase in IL-6, IL-8, TNF-α, and NF-kB mRNA and protein expressions in the uterine tissues of rabbits in their study of IR damage. Again, in different studies, increased IL-1β, TNF-α [22], and TLR4 [23] gene expressions were detected in the ovaries of rats as a result of IR. In the study conducted by Kölükçü et al. [24], it was found that IL-1β, IL-6, and TNF-α levels were significantly suppressed in the group treated with dexmedetomidine in rat uteri exposed to IRI. In our study, statistically significant increases in the expression levels of IL-1β, NF-kB, TLR4 and TNF-α were found in the IRI group (Group 1). Interestingly, a significant decrease was detected in the IL-6 level.

The endocannabinoid system has emerged as a promising novel therapeutic target in inflammatory diseases, cancer types, metabolic, cardiovascular, gastrointestinal diseases and urogenital system diseases [25,26,27,28]. The endocannabinoid system is expressed in the female reproductive system [29]. It has been emphasized that changes in the expression of anandamide, which is an endogenous cannabinoid ligand, and/or CB receptors in the uterus/embryo may be responsible for early pregnancy failure or female infertility [30]. Expression and localization of cannabinoid receptors and enzymes in human oocytes and granulosa cells indicate that the endocannabinoid system plays a role in oocyte maturation [31]. Pagano et al. [32] showed that JWH133 attenuated spontaneous uterine contraction induced by prostaglandin during the diestrus phase, whereas pretreatment with a CB2R blocker abolished the spasmolytic effect of JWH133. This suggested that the mechanism of action of JWH133 was related to the suppression of prostaglandin secretion and synthesis. It has been shown that JWH133 has a strong suppressive effect on the immune system and suppresses experimental autoimmune uveoretinitis in a dose-dependent manner [33]. Moreover, in renal IRI, JWH-133 reduced the level of NF-κB immunoreactivity [13]. The finding that JWH133 down-regulates TLR4 expression in dendritic cells explains the reduced resistance to well-known bacterial, protozoan and viral infections in chronic cannabinoid users [34]. In our study, a significant decrease was found in the IL-1β, IL-6, NF-kB, TLR4, and TNF-α expression levels at a dose of 1 mg/kg JWH-133.

The cannabinoid receptor agonist JWH-133 has been reported in previous studies to exhibit anti-inflammatory effects by targeting various proteins, including IL-1β, IL-6, NF-κB, TLR-4, and TNF-α. JWH-133 is a highly selective full agonist of CBR2, which is expressed in immune cells, making it an attractive therapeutic target for the treatment of inflammatory diseases [5]. Wetlab analysis and docking studies were conducted to investigate the anti-inflammatory activities of JWH-133 on these protein targets. This study discovered that JWH-133 formed a hydrogen bond with the amino acid residues of GLU64 of IL-1β, SER226 of IL-6, and SER62 of TNF-α, its carbonyl (C=O) [35]. This interaction was found to be stabilizing and could potentially inhibit the inflammatory response mediated by these proteins.

Further insights into the binding mode of JWH-133 containing carbonyl functional group revealed that it formed an H-bond network specifically with hydrophobic of SER62, SER226, and GLU64, indicating the potential importance of hydrophobic interactions in mediating the anti-inflammatory effects of JWH-133 on these proteins. Furthermore, the amino acid residues SER62, SER226, and GLU64 are critical to the function of these proteins, and their interaction with JWH-133 suggests that it may exert a significant inhibitory effect on their activity. In addition, JWH-133, with these crucial amino acid interactions, was able to bind with TLR-4 and NF-κB, inhibiting their functions by preventing the formation of key protein–protein interactions necessary for the activation of these pathways. This study, therefore, provides evidence that JWH-133 has promising anti-inflammatory properties by targeting critical proteins involved in the inflammatory response. The lab analysis results, which correspond with the docking studies, further validate the potential of JWH-133 as an effective therapeutic for inflammation-related disorders.

This study has certain limitations. Firstly, since it was a dose-determination study and the effects of JWH-133 on the uterus following IRI were evaluated, potential side effects on other tissues and/or organs were not assessed. However, as the effects of JWH-133 were investigated for the first time in a uterine IRI model, the study focused on addressing preliminary questions. Secondly, this model (3 h ischemia–reperfusion duration) did not fully reflect a real clinical situation. Nevertheless, this duration was sufficient to trigger damage pathways and to explore the effects of potentially beneficial agents.

## 5. Conclusions

The researchers used three different doses (0.2, 1, and 5 mg/kg) of the CB2 receptor agonist JWH-133 in the uterine IR model and demonstrated that all three doses (0.2, 1.5 mg/kg) of JWH-133 decreased the expression levels of IL-1β, IL-6, NF-kB, TLR-4, and TNF-α. The researchers also evaluated the level of interaction between JWH-133 IL-1β, IL-6, NF-κB, TLR-4, and TNF-α at the molecular level through a molecular docking analysis. JWH-133 formed a hydrogen bond with the amino residues of GLU64 of IL-1β, SER226 of IL-6, and SER62 of TNF-α, its carbonyl (C=O). In conclusion, JWH-133 has been shown to exert anti-inflammatory effects by targeting crucial proteins involved in the inflammatory response, including IL-1β, IL-6, and TNF-α, in preventing uterine IR damage. We believe that these data will shed light on future drug analysis studies.

## Figures and Tables

**Table 1:** Expression levels of genes in groups compared to control group.

Gene Symbol	Group 1	Group 2	Group 3	Group 4
	Fold Change	Fold Change	Fold Change	Fold Change
**GAPDH**	1.00	1.00	1.00	1.00
**IL-1β**	3.76 *	0.96	0.70 *	0.53 *
**IL-6**	0.00 *	0.00 *	0.00 *	0.00 *
**NF-κB**	9.85 *	3.51 *	0.88 *	0.50 *
**TLR4**	1.06	0.69 *	0.56 *	0.61
**TNF-α**	3.05 *	1.25	0.70 *	0.49 *

Fold Change (2^(−Delta Delta CT)^) is the normalized gene expression (2^(−Delta CT)^) in the Test Sample divided by the normalized gene expression (2^(−Delta CT)^) in the Control Sample. The *p*-value calculation used is based on parametric, unpaired, two-sample equal variance, two-tailed distribution—a method widely accepted in scientific literature. Fold regulation threshold ≥ 2; * *p* < 0.05; ** *p* < 0.001. (GAPDH: Glyceraldehyde 3-phosphate dehydrogenase, IL-1β: Interleukin-1β, IL-6: Interleukin-6, NF-κB: nuclear factor-kB, TLR4: Toll-like receptor 4, TNF-α: Tumor necrosis factor-α).

**Table 2:** The binding score of each of the proteins with the JWH-133 ligand.

Proteins	Docking Score
IL-1β (PDB-ID: 1L1B)	−3.99
IL-6 (PDB-ID: 1l1R)	−3.08
NF-κB (PDB-ID: 4IDV)	−3.75
TNF-α (PDB-ID: 3L9J)	−3.00
TLR-4 (PDB-ID: 2Z5V)	−4.56

## Data Availability

Original data supporting the findings of this study are available. These data are available upon request from the corresponding author.

## Supplementary Materials

The following supporting information can be downloaded at: https://www.mdpi.com/article/10.3390/life14101214/s1. Figure S1: 3D and 2D interaction of JWH Ligand with IL-1β pocket site (a), IL-6 (b), NF-κB (c), TNF-α (d), TLR-4 (e) respectively, is depicting the hydrogen bond interaction with GLU64, SER226, and SER62.
