# Supplementary material for: The Potential of JWH-133 to Inhibit the TLR4/NF-κB Signaling Pathway in Uterine Ischemia–Reperfusion Injury
Source: Life (Basel). 2024 Sep 24;14(10):1214. doi: 10.3390/life14101214 (PMC11508640; doi:10.3390/life14101214)
Supplement: Supplementary file 1 [file life-14-01214-s001.zip › life-3179616-supplementary.pdf]

Supplementary materials legend

Supplementary materials: 3D and 2D interaction of JWH Ligand with IL-1β pocket site **(a)**, IL-6 **(b)**, NF-κB **(c)**, TNF-α **(d)**, TLR-4 **(e)** respectively, is depicting the hydrogen bond interaction with GLU64, SER226, and SER62.
