# Supplementary figures and images for: The Potential of JWH-133 to Inhibit the TLR4/NF-κB Signaling Pathway in Uterine Ischemia–Reperfusion Injury
Source: Life (Basel). 2024 Sep 24;14(10):1214. doi: 10.3390/life14101214 (PMC11508640; doi:10.3390/life14101214)

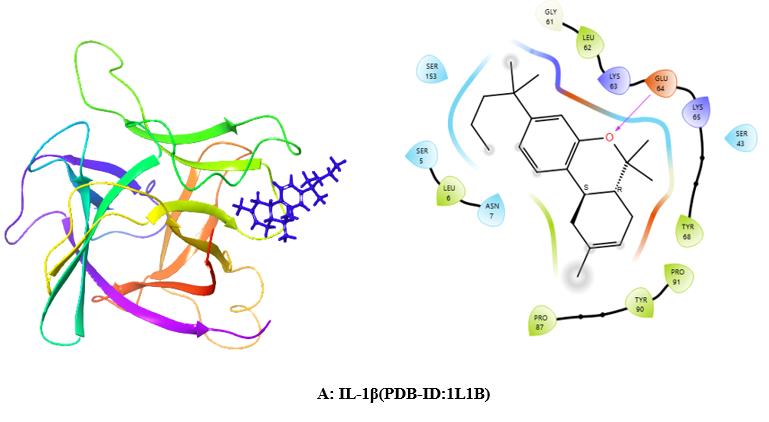

Supplement: Supplementary file 1 [file life-14-01214-s001.zip › Figure 1a.jpg]

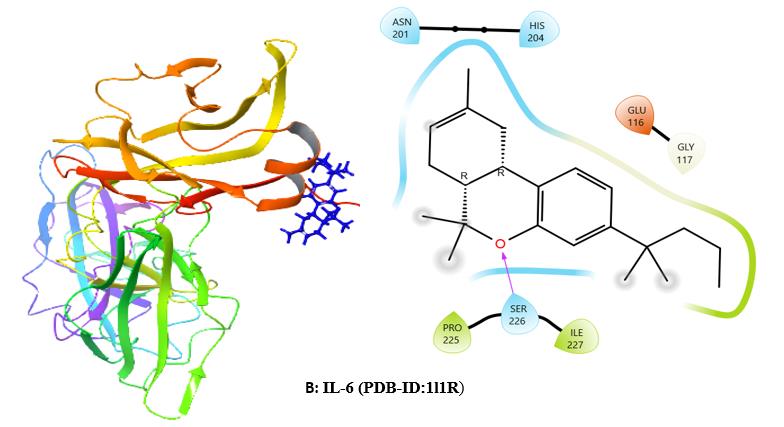

Supplement: Supplementary file 1 [file life-14-01214-s001.zip › Figure 1b.jpg]

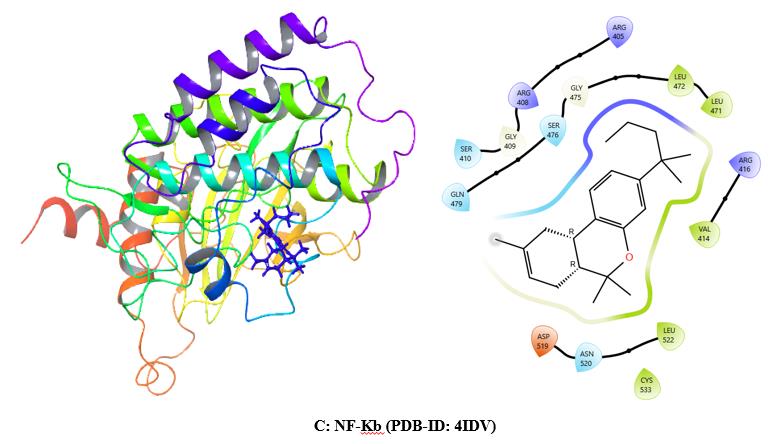

Supplement: Supplementary file 1 [file life-14-01214-s001.zip › Figure 1c.jpg]

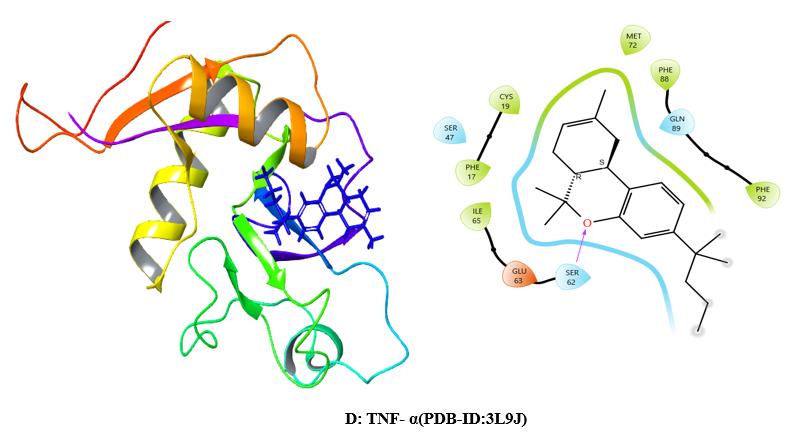

Supplement: Supplementary file 1 [file life-14-01214-s001.zip › Figure 1d.jpg]

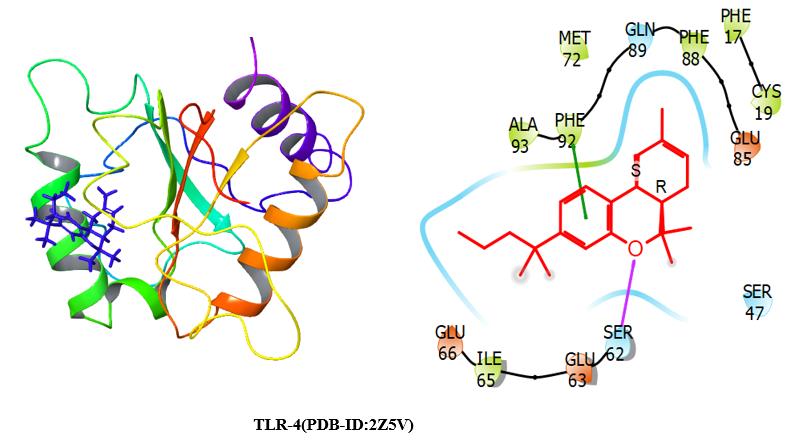

Supplement: Supplementary file 1 [file life-14-01214-s001.zip › Figure 1e.jpg]

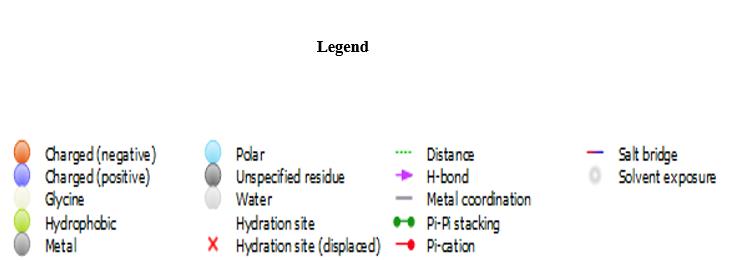

Supplement: Supplementary file 1 [file life-14-01214-s001.zip › Figure 1f.jpg]
